# Sidestream Smoke Extracts from Harm-Reduction and Conventional Camel Cigarettes Inhibit Osteogenic Differentiation via Oxidative Stress and Differential Activation of intrinsic Apoptotic Pathways

**DOI:** 10.3390/antiox11122474

**Published:** 2022-12-15

**Authors:** Nicole R. L. Sparks, Lauren M. Walker, Steven R. Sera, Joseph V. Madrid, Michael Hanna, Edward C. Dominguez, Nicole I. zur Nieden

**Affiliations:** 1 Department of Molecular, Cell & Systems Biology and Stem Cell Center, College of Natural and Agricultural Sciences, University of California Riverside, Riverside, CA 92521, USA; 2 Environmental Toxicology Graduate Program, University of California Riverside, Riverside, CA 92521, USA; 3 Cell, Molecular and Developmental Biology Graduate Program, University of California Riverside, Riverside, CA 92521, USA

**Keywords:** developmental toxicity, embryonic stem cells, osteoblasts, tobacco smoke solution, oxidative stress, hypomineralization

## Abstract

Epidemiological studies suggest cigarette smoking as a probable environmental factor for a variety of congenital anomalies, including low bone mass, increased fracture risk and poor skeletal health. Human and animal in vitro models have confirmed hypomineralization of differentiating cell lines with sidestream smoke being more harmful to developing cells than mainstream smoke. Furthermore, first reports are emerging to suggest a differential impact of conventional versus harm-reduction tobacco products on bone tissue as it develops in the embryo or in vitro. To gather first insight into the molecular mechanism of such differences, we assessed the effect of sidestream smoke solutions from Camel (conventional) and Camel Blue (harm-reduction) cigarettes using a human embryonic stem cell osteogenic differentiation model. Sidestream smoke from the conventional Camel cigarettes concentration-dependently inhibited in vitro calcification triggered by high levels of mitochondrially generated oxidative stress, loss of mitochondrial membrane potential, and reduced ATP production. Camel sidestream smoke also induced DNA damage and caspase 9-dependent apoptosis. Camel Blue-exposed cells, in contrast, invoked only intermediate levels of reactive oxygen species insufficient to activate caspase 3/7. Despite the absence of apoptotic gene activation, damage to the mitochondrial phenotype was still noted concomitant with activation of an anti-inflammatory gene signature and inhibited mineralization. Collectively, the presented findings in differentiating pluripotent stem cells imply that embryos may exhibit low bone mineral density if exposed to environmental smoke during development.

## 1. Introduction

Women make up a staggering 20% of global smokers [1], 10–20% of which continue to smoke during pregnancy [2]. This is despite intensive public health initiatives that have publicized the detrimental effects of tobacco use during pregnancy. Among these are a plethora of neurological and physical developmental defects, including a decrease in overall body length, as well as head and chest circumference [3]. Smoking during any point in pregnancy also associates with derailed bone measurements in the offspring, such as bone age, bone mineral content and density, as well as neonate bone mineral quality [3,4]. Epidemiological studies have shown an increased risk of bone-development malformations, such as cleft lip and palate, and risk of fractures following minor injuries [3,5,6]. 

Despite most of this research surfacing from conventional cigarettes, “harm-reduction” products have become increasingly popular to consumers. As an alternative to conventional cigarettes, pregnant smokers will opt to use cigarettes with lower tar and nicotine content, previously advertised as “light” cigarettes with the assumption that such products are safer to use. However, this perception is concerning as users of harm-reduction products often engage in compensatory smoking to offset reduced nicotine delivery. Consequently, the frequency of smoking-associated cancer deaths seems equally high in those who use harm-reduction products [7]. 

To evaluate the comparative developmental toxicity of harm-reduction and conventional cigarette smoke as it pertains to skeletal malformations that arise upon exposure while in utero our group has recently turned to a well-established osteogenic differentiation model that is based on human embryonic stem cells (hESCs) [8]. Using this model, we have previously evaluated the skeletal embryotoxicity of mainstream (MS) and sidestream (SS) smoke solutions generated from multiple conventional and harm-reduction cigarettes [9]. Comparing MS and SS smoke solutions, our study supported the notion that tobacco has detrimental effects on human osteogenic differentiation with SS smoke, which burns off the tip of the cigarette [10], being more developmentally osteotoxic than MS smoke, which is the smoke actively inhaled by the smoker. Generally, developmental toxicity may be elicited by toxicants due to the cytotoxic nature of the exposure, because the toxicant alters cell fate trajectories in the absence of cytotoxicity [11], or by a combination of both. Intriguingly, sidestream smoke solutions from harm-reduction products elicited differentiation defects at sub-toxic concentrations, while developmental effects for conventional tobacco product solutions followed their general cytotoxicity. This differential effect was strongest for cigarettes of the Camel brand.

We then confirmed the adverse skeletal outcome associated with sidestream smoke encounters in a whole-animal exposure regimen using the zebrafish. At high concentrations, sidestream cigarette smoke from several brands, including Camel, reduced hatching success and overall survival in the developing larvae [12]. At lower, non-lethal concentrations, craniofacial defects were noted that were grounded in impacted cartilage formation and/or bone mineralization independent of whether the skeletal structures were mesoderm or neural crest-derived. Numerous epidemiological, animal and in vitro studies, including ours, therefore now demonstrate the adverse effects of tobacco-related products towards developing embryos and human skeletal health, yet, the molecular underpinnings of the differential adverse skeletal effects caused by harm-reduction products in relation to conventional products remain unclear. 

As one possible molecular initiation event, oxidative stress has recently been discussed to contribute to the embryotoxicity of a variety of different chemicals [13,14,15], including tobacco smoke and its extracts [16]. Arising as a consequence of chemical or environmental insult, oxidative stress is classically defined as the “imbalance of reducing and oxidizing equivalents where the latter predominates” [17]. In such cases, increased production of reactive oxygen species (ROS) contributes to a loss of tissue function [18,19,20,21]. Due to this existing relationship between developmental inhibition and oxidative stress, the purpose of the current study was to evaluate the contribution of oxidative stress to the differential developmental osteotoxicity of Camel smoke solutions observed in vitro.

## 2. Materials and Methods

### 2.1. Cell Culture

Human H9 embryonic stem cells (ESCs), acquired from WiCell (WiCell Research Institute, Madison, WI, USA), were maintained in mTeSR^®^ medium (Stem Cell Technologies, Vancouver, BC, Canada) and kept in the undifferentiated state at 37 °C in a humid 5% CO_2_ environment. Pluripotent colonies were passaged every 5 days by dissociating cells with Accutase^®^ (Innovative Cell Technologies, Inc., San Diego, CA, USA) and a cell scraper. Cells were replated on Matrigel (BD Biosciences, Franklin Lakes, NJ, USA) coated culture plates. 

### 2.2. Osteogenic Differentiation

At confluency, pluripotent colonies were induced to undergo osteogenesis with control differentiation medium consisting of Dulbecco’s modified Eagle’s medium (DMEM; Gibco, Waltham, MA, USA) containing 15% FBS (Atlanta Biologicals, Flowery Branch, GA, USA), 1% non-essential amino acids (NEAA; Gibco), 1:200 penicillin/streptomycin (Gibco), and 0.1 mM β-mercaptoethanol (Sigma-Aldrich, Waltham, MA, USA) for 5 days as described [8]. Starting from the fifth day of culture, control differentiation medium was supplemented for the remaining differentiation duration with osteogenic factors: 0.1 mM β-glycerophosphate (βGP; Sigma-Aldrich), 50 µg/mL ascorbic acid (AA; Sigma-Aldrich), and 1.2 × 10^−7^ M 1.25(OH)_2_ Vitamin D_3_ (VD_3_; Calbiochem, San Diego, CA, USA).

### 2.3. Production of Smoke Solution

Commercially available conventional and harm-reduction Camel cigarettes were purchased from a local retailer and used to make MS and SS smoke solutions with a method described previously in detail [22,23]. Smoke solutions were generated using a University of Kentucky smoking machine that took a 2.2 s puff of MS every minute. MS smoke solution was generated by pulling 30 puffs of MS smoke through 10 mL of DMEM culture medium. During MS smoke production, SS smoke solution was produced by collecting the smoke that burned off the end of the cigarette and pulling it through 10 mL of DMEM. SS smoke was collected continuously, while MS smoke was collected during each puff. Both MS and SS solutions were made at concentrations of 3 puff equivalents (PE). Immediately after preparation, smoke solutions were filtered through a 0.2 micron Acrodisc^®^ PSF Syringe Filter (Pall Corporation, Port Washington, NY, USA), aliquoted into sterile Eppendorf tubes, and stored in a −80 °C freezer until used. Experiments were performed using either MS or SS at previously identified concentrations alongside an untreated control [9]. Specifically, both Camel and Camel Blue MS effective doses were at 1 PE, Camel SS at 0.2 PE and Camel Blue SS at 0.05 PE. All selected effective doses were found to be in the physiological range and thus very relevant to actual human exposure [9]. All non-effective doses were used at 0.01 PE. Osteogenic differentiation of hESCs was induced as described above, and cultures were treated with smoke solution throughout the experiment. Smoke solutions were replenished with each media change. 

### 2.4. Calcium Assay

To determine osteogenic differentiation yield, matrix calcium was quantified by reacting cells lysed in modified radioimmunoprecipitation (RIPA) buffer with Arsenazo III (Genzyme) and subsequent measurement at 655 nm [24]. The concentration of total calcium in the sample was calculated based on a CaCl_2_ standard and normalized to the total protein content of the sample measured with the Lowry method (Bio-Rad, Hercules, CA, USA) [24].

### 2.5. Antioxidant and Caspase Inhibitor Treatment

To counteract tobacco-induced oxidative stress, three antioxidants were used concomitantly with tobacco treatment during days 0–7 of differentiation: ascorbic acid (AA; Sigma-Aldrich) [10 µM], dl-α-tocopherol acetate (vitamin E; Supelco, Sigma-Aldrich) [10 µM], and glutathione reduced ethyl ester (GSH-OEt; Sigma-Aldrich) [500 μM]. The antioxidants were replenished with every medium change.

To explore the involvement of caspases 4, 8 and 9 in tobacco-related inhibition of osteogenic differentiation, tobacco-treated cultures were simultaneously dosed with caspase 4 inhibitor (4i; Z-LEVD-FMK, PromoCell GmbH, Heidelberg, Germany), caspase-8 inhibitor (8i; Z-IETD-FMK, R&D Systems, Minneapolis, MN, USA) or caspase 9 inhibitor (9i; Z-LEHD-FMK, R&D Systems), all at 3 µM, during days 0–7 of differentiation. Inhibitor-supplemented medium was replaced with every medium change.

### 2.6. Superoxide Anion Detection 

Generation of superoxide anion was determined using a Lumimax Superoxide Anion Detection Kit (Agilent Technologies, Santa Clara, CA, USA). H9 cells were trypsinized, washed with phosphate-buffered saline (PBS), and resuspended in fresh medium to incubate for 30 min at 37 °C. A total of 5 × 10^5^ cells were incubated in superoxide anion assay medium including 0.1 mM luminol solution and 125 µM enhancer at room temperature for 30 min. The chemiluminescent light emissions of superoxide anion were measured with a Lucetta™ luminometer (Lonza, Basel, Switzerland).

### 2.7. MitoSOX Assay

Superoxide formation specially produced by mitochondria was assessed using the commercially available MitoSOX Red Mitochondrial Superoxide Indicator dye (ThermoFisher M36008, ThermoFisher, Waltham, MA, USA). Adherent cells were washed with PBS and incubated with 2.5 μM MitoSOX in PBS for 10 min in the dark. Cells were then immediately imaged on a Nikon Ti fluorescent microscope. MitoSOX positive cells were identified using NIH ImageJ analysis software as outlined by Jensen [25].

### 2.8. MitoTracker Staining and Mitochondrial Analysis

Stress-related changes in mitochondrial morphology were visualized and quantified using the MitoTracker Deep Red FM fluorescent dye (ThermoFisher). H9 cells were trypsinized, washed with PBS, and resuspended in 200 nM MitoTracker dye prepared in fresh medium. Cells were incubated in darkness for 20 min at 25 °C, washed with PBS, and fixed in 4% paraformaldehyde for 15 min at room temperature. Fixed cells were then washed three times with PBS and permeabilized with 0.1% Triton X-100 in PBS for 15 min at room temperature. Cells were washed again and counterstained with 1 μg/mL 4’, 6-diamidino-2-phenylindole (DAPI) in PBS for 30 min. Cells were washed three more times with PBS and resuspended in PBS supplemented with 2% FBS and 1 mM ethylenediaminetetraacetic acid (EDTA). Cell number was quantified and adjusted to a concentration of 5 × 10^5^ cells/mL. Cells were spun onto pre-coated Shandon Single Cytoslides (ThermoFisher) using a Shandon Cytospin 3 (Shandon). For each treatment, a 100 µL volume of fixed and stained cellular suspension was loaded into a cytospin funnel and centrifuged at 200 rpm for 5 min at low acceleration/deceleration settings. Slides were allowed to air-dry overnight before mounting with Fluoro-Gel (Electron Microscopy Sciences, Hatfield, PA, USA) for imaging.

Z-stack images were taken using a Leica DMi8 fluorescent confocal microscope and max projected to flatten out each image. Resultant images were pre-processed in ImageJ 1.48v (NIH) to prepare for mitochondrial morphological analysis. The MitoTracker Deep Red channel was first separated from the nuclear DAPI channel to allow for specific analysis of the mitochondria. Images were further processed using the Mitochondrial Network Analysis (MiNA) ImageJ plug-in (https://github.com/ScienceToolkit/MiNA, accessed on 16 November 2021) to prepare images for evaluation of mitochondrial networks within individual cells. Using the default MiNA settings, images were subjected to a 2-pixel gaussian blur rendering through the Enhance Local Contrast median filter, and a final processing through an unsharp mask tool to yield a “skeleton” or tracing of the mitochondrial networks in a given cell. Mitochondrial networks were evaluated using the MiNA analysis method as outlined by Valente et al. [26]. The mitochondrial network length, number of branches, and area of mitochondrial footprint were then automatically measured from the program-generated skeletons to quantify tobacco-related changes to mitochondrial networks. 

### 2.9. Caspase 3/7 Stain

For determination of activated caspases 3/7, cells were incubated for 1 h in a 1X caspase 3/7 reagent conjugated to carboxyfluorescein fluorochrome (Guava Technologies, Hayward, CA, USA). The fluorescent signal was detected in cells where the reagent is covalently bound to the activated caspases, any unbound reagent was washed away with 1X apoptosis buffer provided by the manufacturer. Cells were observed and imaged on a Nikon Ti fluorescent microscope.

### 2.10. Apoptosis RT^2^ Profiler qPCR Array

The correlation between tobacco exposure and apoptosis was examined based on expression changes of 84 apoptosis-associated genes using a Qiagen human Apoptosis RT^2^ Profiler Array (complete gene list accessible at https://geneglobe.qiagen.com/us/product-groups/rt2-profiler-pcr-arrays, accessed on 5 May 2021). For this, hESCs were differentiated into osteoblasts as described above with concomitant exposure to either solvent, non-effective, or effective doses (50% inhibition of calcification) of tobacco smoke solutions as determined from concentration response curves [9]. RNA was isolated using the NucleoSpin RNA kit (Macherey-Nagel, Düren, Germany) and examined for RNA integrity using the Agilent 2100 Bioanalyzer. Only samples with an RNA integrity number of >8 were used for further processing. Five hundred nanograms of RNA were input into a cDNA reaction as described before [8]. qPCR reactions were set up using iQ SYBR Green Supermix (Bio-Rad) and 12.5 ng of cDNA per array well and cycled in a Bio-Rad iQ5 qPCR machine. Data were uploaded to the Qiagen Data Analysis Center at www.SABiosciences.com/pcrarraydataanalysis.php accessed on 5 May 2021, which uses the ΔΔC_T_ method [27] and a two-tailed *t*-test with equal variance for analysis. Normalization was performed to all five housekeeping genes (*ACTB*, *B2M*, *GAPDH*, *HPRT*, *RPLP0*). Heatmaps and volcano plots were made with GraphPad Prism (version 9.4.1, GraphPad Software Inc., San Diego, CA, USA) and VENN diagrams were generated with Venny 2.0 (https://bioinfogp.cnb.csic.es/tools/venny/index.html, accessed on 6 June 2022) [28].

### 2.11. Real-Time Quantitative PCR

Changes in cellular stress-related gene expression related to DNA damage, growth arrest, and apoptosis were assessed using real-time quantitative PCR (qPCR) measurements of *GADD45α*, *GADD45β*, and *GADD45γ* isoform expression. RNA was extracted from cells and subsequently purified using the NucleoSpin RNA kit (Macherey-Nagel) protocol. Isolated RNA was quantified using a NanoDrop^®^ 1000 spectrophotometer (ThermoFisher) at 260 nm. Synthesis of cDNA was performed using 25 ng of total RNA as a template and a cDNA mastermix as described before [8]. Quantitative PCR analysis utilized resultant 25 ng of cDNA transcripts and iQ SYBR Green Supermix (Bio-Rad) on the CFX Connect thermocycler (Bio-Rad). Reactions were programmed for 5 min of initial denaturing at 94 °C, followed by 40 cycles of denaturing at 94 °C for 45 s and annealing at 60 °C for 45 s. The ΔΔC_T_ method [27] was used to calculate n-fold expression in target gene expression by normalizing target C_T_ values to their respective *GAPDH* expression values. Primer sequences for human *GADD45α* were 5’-TTACTCAAGCAGTTACTCCCTACA-3’ and 5’-CCTTCTTCATTTTCACCTCTTTCCA-3’, for *GADD45β* they were 5’-ATGACATCGCCCTGCAAATC-3’ and 5’-GTGACCAGGAGACAATGCAG-3’, and for *GADD45γ* they were 5’-CGCGCTGCAGATCCATTTTA-3’ and 5’-GGGGTTCGAAATGAGGATGC-3’. Primer sequences for human *GAPDH* and *RUNX2* were as described [8]. 

### 2.12. Comet Assay and Analysis

Comet assay slides were prepared prior to cell collection by coating clean frosted microscope slides (ThermoFisher) with 1% normal melting agarose (NMA, Sigma) in PBS. A volume of 75 μL of melted NMA was pipetted directly to the surface of each slide and immediately covered with a coverslip. The NMA layer was allowed to solidify at 25 °C for 10 min prior to coverslip removal. Slides were stored at −20 °C until use. Cells were trypsinized, washed with PBS, counted and resuspended in PBS supplemented with 2% FBS. A cell suspension of 200,000 cells per ml in 0.1% low melting point agarose (LMA, Fisher Bioreagents, Pittsburgh, PA, USA) was prepared immediately prior to distributing cells onto slides for the assay. NMA-coated slides were allowed to come to room temperature before coating with the LMA-cell mixture. The cell-LMA agarose solution was pipetted directly onto each slide in a volume of 75 μL and immediately covered with a coverslip. The LMA-cell layer was allowed to solidify at 25 °C for 10 min prior to coverslip removal. An additional 5 min solidification period at 25 °C was observed after the coverslip was removed. The slides were then placed in a slide tray and the cells were lysed in comet assay lysis buffer (1.2 M NaCl, 100 mM Na2EDTA, 0.1% sodium lauryl sarcosinate, 0.26 M NaOH, pH > 13) for 1 h at 25 °C. Next, lysis buffer was aspirated off the slides and replaced with electrophoresis solution (0.03 M NaOH, 2 mM Na2EDTA, pH~12.3) for 20 min at 4 °C. Slides were transferred to an electrophoresis box and subjected to electrophoresis for 20 min at 1 V/cm and 4 °C. Slides were rinsed in ddH_2_O for 1 min, then immersed in 70% ethanol for 5 min. Slides were air-dried overnight and stained with 1 μg/mL DAPI in PBS for 30 min. 

Slides were rinsed with PBS and air dried prior to imaging on a Nikon Ti Eclipse fluorescent microscope. Three slides per treatment group were prepared and scored visually. Fifty cells per slide were scored. Fluorouracil (5-FU; Sigma-Aldrich) treated cells were evaluated as a positive control. To ensure accuracy of comet parameter measurements, DAPI signal intensity was subjected to a color threshold in ImageJ (commands used: Image > Adjust > Color Threshold).

Cells were assessed visually according to comet tail length and Olive tail moment. Comet tail length was manually quantified in ImageJ by measuring the distance spanned by comet tails (if present) from the edge of the cell nucleus to the furthest end of the comet tail. Olive tail moment (OTM) was determined by multiplying the distance between the center of the comet head and tail and the percentage of total DNA in the comet tail as previously described.

Cells were designated as class 0 (undamaged cell; 0–10% of DNA in comet tail) while cells with tails were ranked according to increasing tail size: class 1 (minimally damaged; 10–20% of DNA in comet tail), class 2 (moderately damaged; 20–40% of DNA in comet tail), class 3 (severely damaged; 40–60% of DNA in comet tail), and class 4 (maximally damaged; 70–90% of DNA in comet tail).

### 2.13. Western Blotting

Prior to lysing, cells were pretreated for 30 min with 1 mM sodium orthovanadate to inhibit protein tyrosine phosphatases. Cells were then lysed with RIPA buffer (pH 7.4 150 mM NaCl, 2 mM EDTA, 50 mM Tris-HCl pH 7.4, 1% NP-40, 0.5% sodium deoxycholate, 0.1% sodium dodecyl sulfate (SDS), 1 mM sodium orthovanadate, 1 mM sodium fluoride, 1 mM phenylmethylsulfonyl fluoride (PMSF), and 1:100 Halt Protease Inhibitor Cocktail (ThermoFisher) to collect whole-cell protein lysates. A modified Lowry protein assay (Bio-Rad DC™ protein assay) was used to determine protein concentration in fresh lysates. For Western blot analysis, equal amounts of protein per treatment group were loaded into a 6–10% SDS/polyacrylamide gel and separated by electrophoresis prior to electrophoretic transfer to a polyvinylidene difluoride (PDVF) membrane. Membranes were immediately blocked in 5% bovine serum albumin (BSA) in tris-buffered saline with tween 20 (TBS-T) for 30 min at room temperature on an orbital shaker. Membranes were incubated with one of the following primary antibodies (1:1000) for 2 h at room temperature with shaking: mouse anti-caspase 8 (CST 9746S), mouse anti-caspase 9 (CST 9508S), rabbit anti-phospho-caspase 9 (Tyr153) (abcam ab79202), rabbit anti-c-Abl (CST 2862S), rabbit anti-phospho-c-Abl (Tyr245) (CST 2868S), rabbit anti-phospho-c-Abl (Tyr412) (CST 247C7S), rabbit anti-caspase 4 (ab22687) mouse anti-actin (CST 3700S). Membranes were subsequently incubated for 1 h at room temperature with horseradish peroxidase-conjugated anti-rabbit (CST 7074S) or anti-mouse (CST 7076S) secondary antibody (1:2000). Bands were visualized using chemiluminescence substrate (SuperSignal West Pico PLUS Chemiluminescent Substrate, ThermoFisher) and the Bio-Rad ChemiDoc MP System imager.

### 2.14. Live/Dead Assay

Healthy live and apoptotic cell populations were quantified using the LIVE/DEAD Viability/Cytotoxicity Kit (ThermoFisher L3224). H9 cells were trypsinized, washed with PBS, and resuspended in fresh medium containing 0.1 µM calcein AM and 8 µM ethidium homodimer-1 (EthD-1) as described [29]. Cells were incubated away from light for 20 min at 25 °C, washed with PBS, and centrifuge-strained to encourage a single-cell suspension (Fisher Scientific, 08-771-23). Cells were resuspended in ice-cold PBS supplemented with 2% FBS and immediately analyzed on a FACSCalibur Flow Cytometer (BD Biosciences). Fluorescence was detected at excitation/emission at λ = 494/517 nm and 517/617 nm. Cytometer gating was set using unstained untreated samples and adjusting forward scatter and side-scatter light. For each sample, 10,000 events were collected.

### 2.15. Mitochondrial Membrane Potential

Changes in mitochondrial membrane potential were assessed using the commercially available JC-1 Dye (ThermoFisher T3168). Cells were trypsinized, washed with PBS, and resuspended in fresh medium containing 5 µM JC-1. Cells were incubated for 20 min at 25 °C, washed with PBS, and centrifuge-strained to break up cells into a single-cell suspension (Fisher Scientific, 08-771-23). Cells were subsequently resuspended in ice-cold PBS supplemented with 2% FBS and immediately analyzed on a FACSCalibur Flow Cytometer (BD Biosciences). Fluorescence was detected at excitation/emission at λ = 488/530 nm and 488/585 nm. Cytometer gating was set using unstained untreated samples and adjusting forward scatter and side-scatter light. For each sample, 10,000 events were collected.

### 2.16. ATP:AMP Assays

ATP and AMP levels were quantified via an ATP Determination Kit (ThermoFisher A22066) and an AMP ELISA (Kamiya Biomedical Company KT-52769, Seattle, WA, USA), respectively. For both assessments, cells were washed with PBS, trypsinized and resuspended in PBS. 

In the ATP assay, a cell suspension for each treatment group was counted and readjusted to a final concentration of 12.5 × 10^6^ cells/mL. For each reading, 10 µL of ATP standard or sample was combined with 90 µL of the ATP reaction solution provided by the ATP Determination Kit immediately before to recording the reaction luminescence output using a Lucetta™ luminometer (Lonza). Relative ng amounts of ATP in each treatment group were determined from an ATP standard curve.

For AMP determination, each cell suspension was adjusted to a final concentration of 1.25 × 10^6^ cells/mL. PBS-suspended cells were prepared for ELISA analysis by freeze–thawing three times with gentle mixing between freezing followed by centrifugation at 1000× *g* for 15 min at 4 °C. Cell lysates were assessed according to the manufacturer’s ELISA protocol and final optical density was measured at 450 nm using an iMark microplate reader (Bio-Rad). Relative ng amounts of AMP were determined using a standard curve constructed from an AMP standard.

### 2.17. Statistical Analysis

Assay results were statistically assessed with a paired student’s *t*-test or one-way Analysis of Variance (ANOVA) as appropriate (GraphPad Prism 9.4.1, GraphPad Software Inc.). Normality and equal variance were examined with a Shapiro–Wilk test and Bartlett’s test, respectively. Non-normal data were log transformed before proceeding to a one-way ANOVA to compare the mean of each treatment with the mean of the control group. A Dunnett’s test was used to correct for multiple comparisons. For all conducted tests, *p*-values below 0.05 were considered significant.

## 3. Results

### 3.1. Embryotoxicity of SS Smoke Extract Is Associated with Oxidative Stress

**Figure 1:**
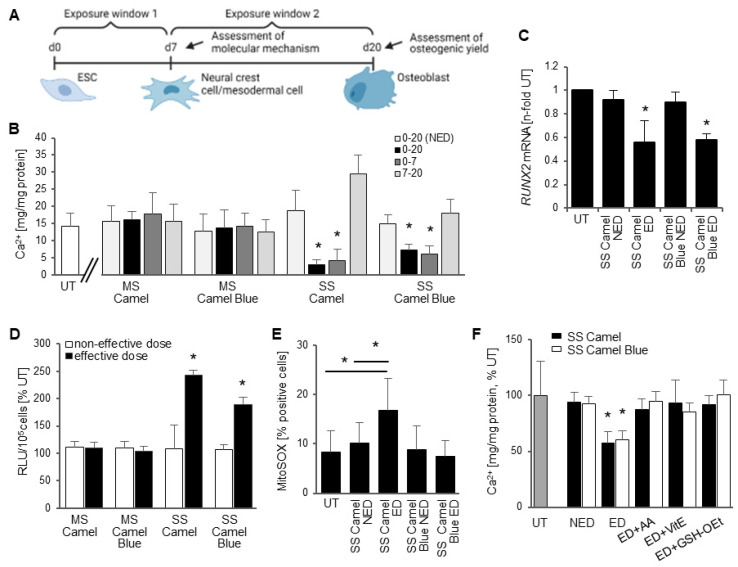
Differentiation inhibition caused by harm-reduction tobacco exposure occurred through intermediate levels of reactive oxygen species. (**A**) Schematic of experimental design and exposure scheme. Created with BioRender.com. (**B**) Mineralization assay using Arsenazo III to determine osteogenic differentiation efficiency after exposure to previously identified effective doses of Camel cigarette smoke extracts [9] within different time windows. (**C**) *RUNX2* mRNA expression measured with qRT-PCR, normalized to *GAPDH* and compared to the untreated control, set at 1; *n* = 3 ± SD. (**D**) Superoxide anion content measured upon reaction of the cells with luminol and charted as percent of the untreated cultures; d7, *n* = 3 ± SD. (**E**) Cells were exposed for seven days, incubated with MitoSOX, photographed and positive cells counted. Only Camel exposure elicited a significant increase specifically in mitochondrial oxidative stress. (**F**) Calcium deposit was quantified from cultures exposed for 20 days with and without concomitant addition of antioxidants. Effective doses of tobacco smoke solutions and extracts reduced calcification, which was rescued with antioxidant treatment; *n* = 3 ± SD. * *p* < 0.05, One-Way ANOVA versus untreated cultures. AA, ascorbic acid; ED, effective dose; GSHOEt, glutathione reduced ethyl ester; MS, mainstream; NED, non-effective dose; RLU, relative light unit; SS, sidestream; UT, untreated solvent control; VitE, Vitamin E.

We have previously determined that Camel and Camel Blue smoke solutions impede osteogenic differentiation of hESCs when added throughout the differentiation period from d0 to d20 [9]. While this exposure regimen is commonplace in developmental toxicity assays that rely on ESCs [30,31,32,33,34,35], it does not illuminate the critical window of susceptibility to certain chemicals. Yet, knowing the window of susceptibility may allow a first insight into the molecular mechanism of toxicity and the potential identity of the target cell or tissue. Therefore, in the current study we performed an initial experiment in which we dosed cells with an effective dose, determined from a concentration-response curve as the puff equivalent concentration that reduced calcification to 50% [9], and a non-effective dose (no effect), separating the differentiation process into two exposure windows (Figure 1A). 

Camel and Camel Blue SS smoke solutions elicited a differentiation defect in the first exposure window (d0–7) that encompasses exit from pluripotency and the specification of cells with osteogenic potential (Figure 1B), but not in the second exposure window (d7–20), which represents the maturation of the osteoprogenitors. By day seven, cells have differentiated into neural crest and mesodermal progenitors that both have the potential to generate mesenchymal cells that continue to differentiate and mature into osteoblasts [8]. Based on this result, it could be speculated that the differentiation defect observed after exposure to tobacco smoke might stem from an error in the early commitment of cells with osteogenic potential rather than from interference with the maturation process. This notion was exemplified by a reduction in the mRNA of *RUNX2*, a master regulatory transcription factor of the osteogenic lineage, in tobacco treated cells measured on d7 (Figure 1C).

To determine the contribution of oxidative stress to the differential developmental osteotoxicity observed between harm-reduction and conventional Camel cigarette smoke solutions, we next investigated the level of superoxide anion (O_2_^•−^) that had been generated seven days into exposure. MS Camel and MS Camel Blue showed no statistical difference in O_2_^•−^ content, while the Camel SS smoke extract revealed a 2.4-fold increase in the effective dose over the non-effective dose (Figure 1D). In contrast, the Camel Blue SS effective dose evoked elevated O_2_^•−^ content in the range between 1.6- and 1.9-fold. Further evaluation of the potential source of the O_2_^•−^ uncovered elevated mitochondrial oxidative stress in the Camel SS, but not in Camel Blue SS extract also on day 7 (Figure 1E). Treatment with antioxidants during smoke extract exposure equalized O_2_^•−^ content to solvent controls (Supplemental Figure S1) and rescued calcification in both effective doses (Figure 1F), causally implicating oxidative stress in the observed osteogenic defect.

### 3.2. Camel SS and Camel Blue SS Smoke Differentially Alter Oxidative Stress Associated Transcripts

**Figure 2:**
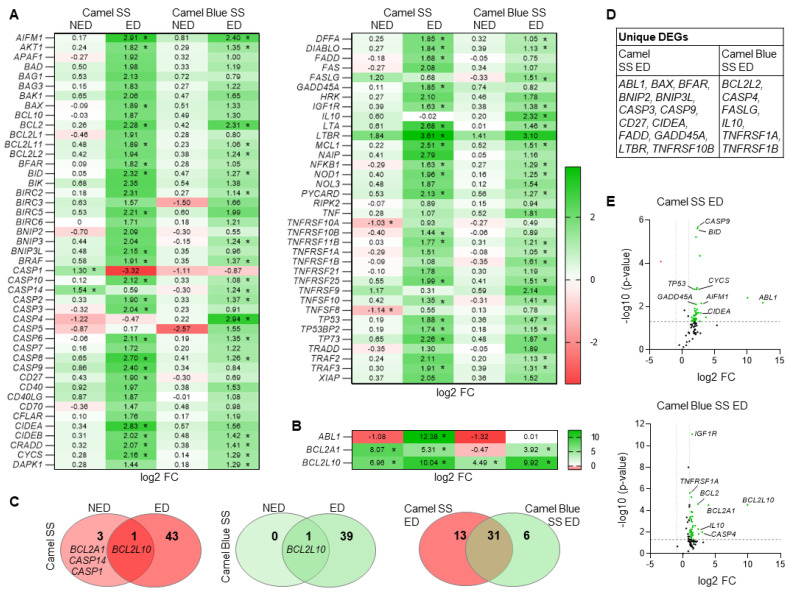
Medium throughput transcript analysis reveals common and differentially expressed apoptosis-related mRNAs in hESCs exposed to conventional and harm-reduction Camel smoke extracts. (**A**,**B**) Heat maps of apoptotic mRNAs de-regulated in tobacco exposed hESCs as measured with the RT^2^ qPCR array for apoptosis. * *p* < 0.05 vs UT. (**C**) VENN diagrams showing relations between differentially expressed transcripts in dependence of cigarette smoke extract type (log_2_FC > 1; Adj. *p*-value < 0.05). Venn diagrams were generated with Venny 2.0 [28]. (**D**) Table identifying the transcripts uniquely regulated by the two cigarette smoke extracts. (**E**) Volcano Plot for Camel SS ED and Camel Blue SS ED exposed cells. ED, effective dose; FC, fold change; NED, non-effective dose; SS, sidestream; UT, untreated solvent control.

In an effort to link oxidative stress-mediated embryotoxicity to altered gene regulation, we next performed a Qiagen RT^2^ Apoptosis Profiler qPCR array (Figure 2). A heatmap generated from all transcripts across all treatment groups indicated a close relationship between the untreated control, Camel SS and Camel Blue SS non-effective doses (Figure 2A,B), with only minimal changes in gene expression patterns between these groups. In contrast, global apoptotic gene regulation was significantly different in the effective doses of Camel SS and Camel Blue SS smoke extract (Figure 2C) with both sharing 31 differentially regulated mRNAs. In line with the elevated O_2_^•−^ levels measured in the effective doses, mRNAs involved in ROS signaling were highly expressed in the effective dose of Camel SS smoke extract and to a lesser extent in the effective dose of Camel Blue SS. These same mRNAs were mainly unaltered in the non-effective doses (Figure 2C). Volcano plots further underscored the differential regulation of additional mRNAs not shared between the two smoke solutions (Figure 2D,E) pointing to alternate mechanisms of toxicity.

### 3.3. Conventional Camel SS, but Not the Harm-Reduction Camel Blue SS Smoke Extract Elicits Apoptotic Gene Expression and Activates Executioner Caspases

**Figure 3:**
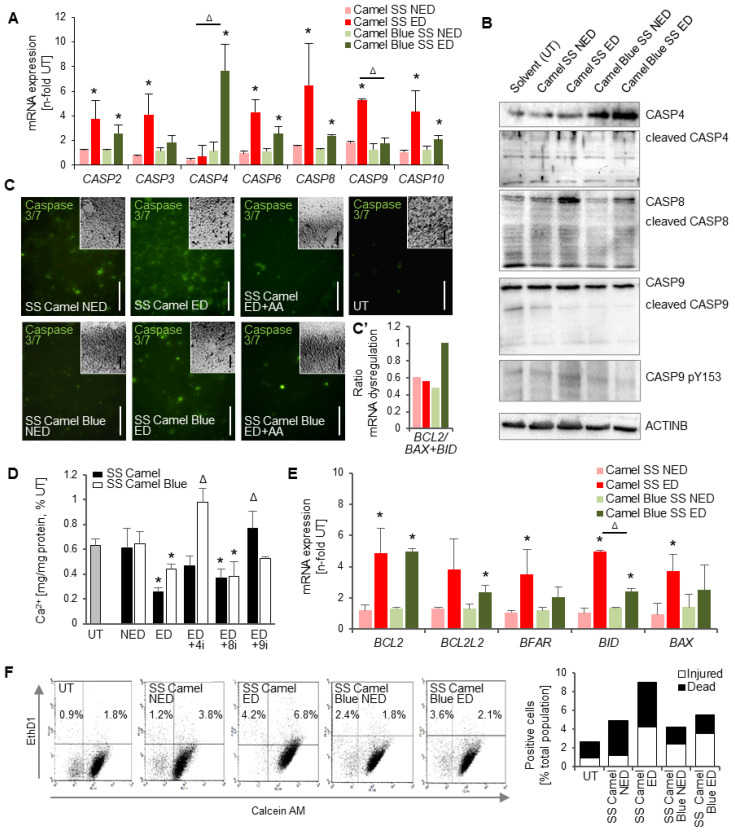
Camel Blue SS elicits a weaker apoptosis response than Camel SS. (**A**) RT^2^ qPCR array for apoptosis identified distinct expression patterns of various caspase isoforms between Camel and Camel Blue SS smoke exposed cells. *n* = 3 ± SD. * *p* < 0.05, two-tailed *t*-test. ^Δ^ *p* < 0.05, two-tailed *t*-test between Camel SS ED and Camel Blue SS ED. (**B**) Western blots revealed the differential activation of caspases associated with extrinsic and intrinsic apoptotic pathways. (**C**) Accordingly, the executioner caspases 3/7 were highly activated in cells exposed to Camel, but only mildly when exposed to Camel Blue. Antioxidant treatment inhibited this activation. Insets show brightfield images of the same field of view. Bar = 100 µM. (**C’**) The ratio of *BCL2* to *BAX* mRNA expression suggested an anti-apoptotic response in Camel Blue SS cultures. (**D**) Inhibition of these caspases rescued calcification in cells treated with effective doses of tobacco products; *n* = 5 ± SD. * *p* < 0.05, One-Way ANOVA versus untreated cultures. ^Δ^ *p* < 0.05, One-Way ANOVA versus ED. (**E**) Some proapoptotic genes were found upregulated in both Camel and Camel Blue SS cultures. *n* = 5 ± SD. * *p* < 0.05, two-tailed *t*-test. ^Δ^ *p* < 0.05, two-tailed *t*-test between Camel SS ED and Camel Blue SS ED. (**F**) LIVE/DEAD assay revealed cell death in cells exposed to conventional smoke extracts only. 4i, caspase 4 inhibitor; 8i, caspase 8 inhibitor; 9i, caspase 9 inhibitor; AA, ascorbic acid; ED, effective dose; NED, non-effective dose; SS, sidestream; UT, untreated solvent control.

The high O_2_^•−^ levels found in Camel SS effective doses cultures occurred in the presence of up-regulated *CASP9* mRNA (Figure 3A), a transcript identified to be uniquely regulated in Camel SS ED (Figure 2D). Respective Western blots with an antibody against caspase 9 specifically phosphorylated at Y153, a well-established activation mark [36], detected increased levels in the Camel SS effective dose (Figure 3B). Concomitantly, and in line with the unique regulation of *CASP3* mRNA (Figure 2D), downstream executioner caspases 3 and 7 were found to be highly activated in Camel SS (Figure 3C). In contrast, the intermediate intracellular O2^•−^ levels released upon exposure to Camel Blue SS were associated with marginal activation signals observed in the caspase3/7 stain (Figure 3C). Indeed, *CASP9* mRNA was not significantly elevated (Figure 3A). Conversely, however, caspase 4 showed higher mRNA and protein expression unique to Camel Blue SS (Figure 2D and Figure 3A,C). 

Inhibition of caspase 9 in Camel SS effective doses and inhibition of caspase 4 in Camel Blue SS effective doses rescued calcification, providing an isoform-specific causal link between caspase activation and differentiation inhibition for both Camel products (Figure 3D). Of note, *CASP8* mRNA (Figure 3A) as well as higher levels of total and cleaved caspase 8 protein expression (Figure 3B) were also noted for Camel SS and Camel Blue SS, and, although higher in Camel SS than Camel Blue SS, were not followed further due to the dual activation by both tobacco products. 

Changes in mRNA expression were also observed for other apoptosis-related genes (Figure 3E). Significant upregulation of mRNA expression for the pro-apoptotic factor *BID* was observed in both effective doses, while *BAX* up-regulation was specific to the Camel SS effective dose. Anti-apoptotic genes *BCL2L10*, *BCL2A1*, and *BCL2* were also upregulated in both Camel SS and Camel Blue SS effective doses (Figure 2B and Figure 3E). However, the anti-apoptotic gene *BCL2A1* was highest in the SS Camel non-effective dose—potentially explaining the survival noted in those cultures. Similarly, despite the activation of multiple apoptotic genes, the ratio between *BCL2* and *BAX/BID* was the most beneficial for survival in the effective dose of SS Camel Blue (Figure 3C’).

Indeed, an increased Bcl2/Bax ratio has been suggested to act as an early protective response to apoptotic stimuli, since the enhanced expression of the anti-apoptotic protein Bcl2 can counteract the rising Bax expression [37]. In line with this notion, a Live/Dead assay revealed Camel SS effective dose cultures to possess the highest percentage of dead cells, while the Camel Blue SS exposed cultures had a lower percentage of fully dead cells (Figure 3F). Both, however, had a similar number of injured cells (Figure 3F). 

### 3.4. Conventional Camel, but Not the Harm-Reduction Camel Blue Smoke Solution Elicits a DNA Damage Response

**Figure 4:**
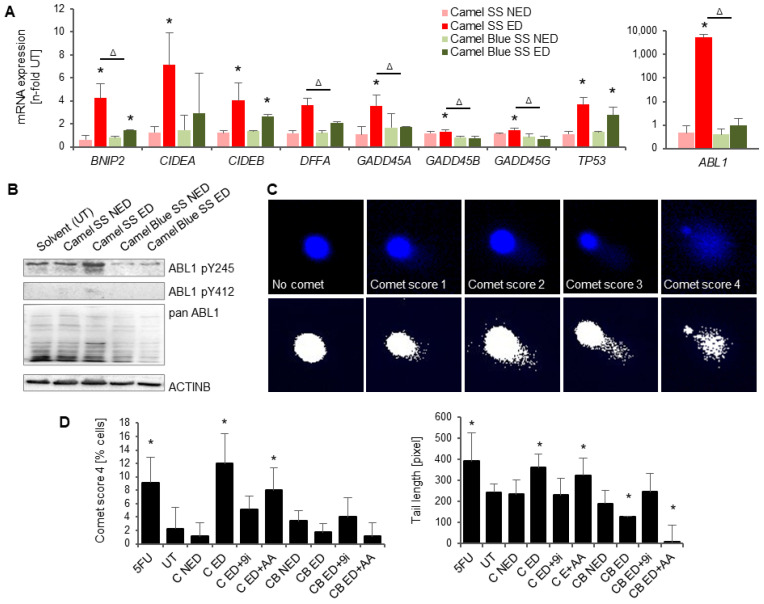
Reduced viability in hESCs exposed to conventional Camel extract is due to DNA damage. (**A**) RT^2^ qPCR array for apoptosis found upregulation of genes associated with DNA damage response in Camel SS smoke exposed cells. *n* = 3 ± SD. * *p* < 0.05, two-tailed *t*-test versus UT, ^Δ^ *p* < 0.05, two-tailed *t*-test between Camel SS ED and Camel Blue SS ED. (**B**) Western blots confirmed ABL1 activation in Camel SS effective doses at the protein level. (**C**,**D**) Comet assays confirm DNA damage in response to Camel exposure, which was absent in Camel Blue exposed cells and cells treated with antioxidant. *n* = 3 ± SD. * *p* < 0.05, One-Way ANOVA versus untreated cultures. Scale bar = 63×. 9i, caspase 9 inhibitor; AA, ascorbic acid; ED, effective dose; NED, non-effective dose; SS, sidestream; UT, untreated solvent control.

The noted severity of apoptosis in the Camel SS effective dose may also be caused by an upregulation in genes associated with genotoxicity response. Indeed, the cell cycle arrest and DNA damage genes *GADD45α*, *GADD45β*, *GADD45γ*, *BNIP2*, and *DFFA* mRNA were significantly upregulated exclusively in cells treated with a Camel SS effective dose (Figure 4A). 

Furthermore, mRNA for the DNA-damage response kinase *ABL1* was only upregulated in the Camel SS effective dose and was notably the highest regulated gene observed (Figure 2B and Figure 4A). Western blot analysis confirmed greater ABL1 phosphorylation at Y412 and Y245 (Figure 4B), residues that contribute to full kinase activation in the event of DNA damage and subsequent DNA repair response [38,39]. 

Additional mRNA markers related to cellular stress, *CIDEA*, *CIDEB*, and *TP53* were also examined. *CIDEB* mRNA was upregulated in both Camel SS and Camel Blue SS effective doses while significance could only be established for *CIDEA* mRNA in Camel SS treated cultures. *TP53* mRNA levels were conspicuously upregulated for Camel SS and Camel Blue SS effective doses, suggesting adverse molecular misregulation by both treatments. 

Confirmatory assessment for DNA lesions was then performed using a Comet Assay. Camel SS effective dose exposed cells demonstrated a significantly higher proportion of the severely damaged comet phenotype and larger DNA lesion tails (Figure 4C,D), an effect not observed in the Camel Blue SS effective dose. Co-treatment with a caspase 9 inhibitor reduced observed comet severity in Camel SS smoke extract-treated cells. As apoptosis-driven DNA fragmentation can be visualized as comets in the comet assay, it is possible that caspase 9 inhibitor treatment reduced comet severity by preventing caspase 9 mediated apoptotic signaling. Ascorbic acid supplement of Camel SS cultures did not reduce comet severity, suggesting that antioxidant treatment was not efficient to prevent DNA damage during the exposure period despite lowering O_2_^•−^ content and rescuing osteogenesis (compare Figure 1F and Supplemental Figure S1).

### 3.5. Camel and Camel Blue Damage Mitochondria with Differential Severity

**Figure 5:**
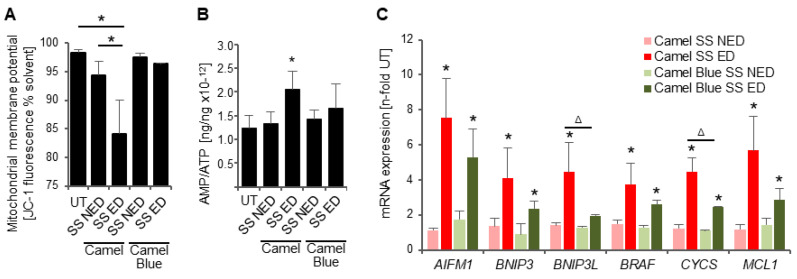
Deterioration of mitochondrial health in exposed hESCs. (**A**) Mitochondrial membrane potential measurements revealed a reduced membrane potential in Camel exposed cells as a sign for execution of the intrinsic apoptotic pathway. * *p* < 0.05, One-Way ANOVA versus untreated or NED cultures (**B**) AMP-to-ATP ratio was increased in Camel SS ED, suggesting mitochondrial dysfunction. * *p* < 0.05, One-Way ANOVA versus untreated cultures. (**C**) qPCR array analysis revealed upregulation of mRNAs associated with integral mitochondrial apoptosis in both Camel SS and Camel Blue SS ED. * *p* < 0.05, two-tailed *t*-test versus untreated control, ^Δ^ *p* < 0.05, two-tailed *t*-test between Camel SS ED and Camel Blue SS ED. ED, effective dose; NED, non-effective dose; SS, sidestream; UT, untreated solvent control.

Since high levels of mitochondria-specific O_2_^•−^ were observed in Camel SS effective dose cultures, mitochondrial health and morphological parameters were next evaluated to determine if mitochondrial dysfunction was also a factor in observed osteogenic inhibition. Mitochondrial membrane potential was significantly reduced in the Camel SS effective dose, which suggested a disturbance to mitochondrial function (Figure 5A). In contrast, mitochondrial membrane potential in Camel Blue SS effective doses remained unchanged from the untreated group. Further, comparative analysis of cellular AMP and ATP levels revealed that the AMP/ATP ratio was increased in the Camel SS effective dose (Figure 5B). This outcome signifies a potential underproduction of ATP and further evidence of mitochondrial dysfunction in Camel SS treated cells.

Additional evidence to support this notion came from the qPCR array. *BRAF*, *BNIP3* and *CYCS* were upregulated by the Camel SS effective dose, while only a mild upregulation was observed in the Camel Blue SS effective dose (Figure 5C). In addition, *BNIP3L* was exclusively upregulated in Camel SS effective dose only (Figure 5C, compare Figure 2D). Given that the latter two proteins are involved in permeabilization of the mitochondrial outer membrane to prepare for cytochrome C release [40,41,42,43,44], it follows that they could have caused the mitochondrial membrane potential depolarization in Camel SS cultures. Collectively, these results suggest a strong mitochondrial-driven apoptotic response in Camel SS cultures. However, the similar *AIFM1* expression patterns between both Camel and Camel Blue SS indicates a potential issue with mitochondrial function was not only occurring in Camel SS smoke extract, but in Camel Blue SS smoke extract also. 

**Figure 6:**
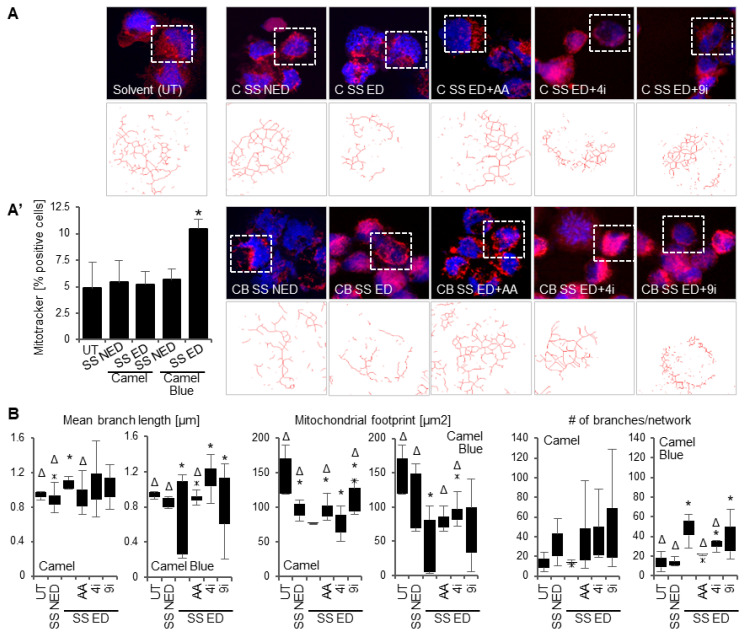
Tobacco smoke exposure elicits changes in mitochondrial networks. (**A**) MitoTracker and MiNA visualization of mitochondrial networks, magnification 63×. (**A’**) MitoTracker dye analysis revealed increased mitochondrial signal in the Camel Blue SS effective dose. * *p* < 0.05, One-Way ANOVA versus untreated cultures. (**B**) Changes to mitochondrial networks were assessed via mean branch length, mitochondrial footprint, and branches per network. * *p* < 0.05, One-Way ANOVA versus untreated cultures, ^Δ^ *p* < 0.05, One-Way ANOVA versus ED, x denotes max or min outliers. 4i, caspase 4 inhibitor; 9i, caspase 9 inhibitor; C, Camel; CB, Camel Blue; ED, effective dose; NED, non-effective dose; SS, sidestream; UT, untreated solvent control.

Quantification of mitochondrial number from confocal images of Mitotracker stained cells found that the total mitochondrial population in the Camel SS effective dose did not deviate from the untreated (Figure 6A’), despite the altered mitochondrial function that was measured in this group. Cells treated with Camel Blue SS, conversely, featured a significant increase in total mitochondrial “bright spots”. Collectively, these outcomes infer that Camel SS and Camel Blue SS elicited differential responses in mitochondrial behavior, which validated investigating these outcomes in further detail.

Mitochondrial networks were characterized by measuring mitochondrial network branch lengths, branch numbers per network, and overall mitochondrial footprint. Cells exposed to Camel SS showed reduced network interconnection (Figure 6A) as well as a significant increase in mitochondrial branch length and a decrease in the overall mitochondrial footprint (Figure 6B). Concurrent treatment of Camel SS effective doses with an inhibitor to caspase 9, the caspase activated to a higher extent in this condition, but not to caspase 4, restored network morphology (Figure 6A), and rescued mitochondrial footprint (Figure 6B). Camel Blue SS exposed cells, in contrast, demonstrated network branch lengths that were not significantly different from the untreated cultures (Figure 6B). Trends towards decreased branch lengths were detectable, however. Cells exposed to Camel Blue SS also demonstrated more highly branched networks and a significantly reduced mitochondrial footprint.

Simultaneous treatment of Camel Blue SS effective doses with caspase 9 inhibitor yielded abnormal mitochondrial network morphology that was comprised of shorter fragmented networks mixed with some filamentous, interconnected networks (Figure 6A). Caspase 9 inhibitor treatment did not significantly rescue mean branch length or mitochondrial footprint (Figure 6B), suggesting a lack of intrinsic mitochondrial apoptosis response. Treatment with caspase 4 inhibitor did, however, restore interconnected mitochondrial network morphology (Figure 6A), rescued mitochondrial footprint (Figure 6B) and decreased the number of branches per network (Figure 6B) suggesting caspase 4 involvement in aberrant mitochondrial behaviors under Camel Blue SS treatment. 

Cellular redox status also appeared to partly influence mitochondrial network morphology in both Camel SS and Camel Blue SS effective doses. Concurrent treatment with antioxidant ascorbic acid rescued branch length and slightly restored mitochondrial footprint in Camel SS cultures (Figure 6B). Camel Blue SS effective doses with ascorbic acid reestablished interconnected mitochondrial network morphology (Figure 6A) and modestly restored the mean branch length range by reducing the incidence of short branch fragments (Figure 6B) implicating a causal relationship between oxidative stress and mitochondrial health. 

## 4. Discussion

Tobacco smoke has been shown to reduce bone density and increase osteopenia and osteoporosis rates in adults [45]. Due to the shortage of information on toxic effects of tobacco smoke on the developing skeleton, we previously used hESCs directed through an osteogenic lineage to assess the potency of tobacco products to inhibit calcification in differentiating osteogenic cultures [9]. Based on this endpoint, differential embryotoxicity was observed in cultures treated with cigarette smoke solutions from MS and SS smoke of Camel and Camel Blue cigarettes. Indeed, other studies found that SS smoke from conventional cigarettes was more potent than MS smoke, in very diverse endpoints such as free radical species levels, angiogenesis, oviductal function, adverse IVF outcome, sperm motility, and attachment ability of peri-implantation embryonic cells [46,47,48,49,50,51,52,53]. Regarding differentiating osteoblasts, our previous study found that conventional MS smoke did not hinder the viability of developing osteoblasts or their differentiation [9]. However, SS smoke always showed detrimental effects at lower concentrations than MS smoke and inhibited both calcification and the survival of the osteogenic cultures. We extend this finding here to a specific susceptibility window that does not include maturation events, but rather represents an early window of development in which cells commit and specify. During this early time window, only SS smoke elicited oxidative stress, but not MS smoke. 

Since it has not been unequivocally answered whether harm-reduction products confer reduced harm on differentiating osteogenic cells, in the current study, smoke extract from a harm-reduction cigarette was compared to a conventional cigarette. Our data show exposure to Camel Blue SS smoke demonstrated differentiation inhibition in developing osteoblasts at sub-cytotoxic concentrations. This enhanced potency of the harm-reduction SS smoke may be explained by the alteration in chemical composition associated with the process of lowering tar and nicotine content during manufacturing. During this process, other constituents found in the complex chemical blend of cigarette smoke such as nitrate, nitrogen oxide, and tobacco-specific nitrosamines may be enriched [54]. Individually, these chemicals can cause adverse health effects in mammalian cells [55,56,57,58]. Not only can concentrations of such chemicals be higher in harm-reduction cigarettes because of the processing required to reduce content of other carcinogens, but smokers also overcompensate for delivered nicotine by smoking more cigarettes or by inhaling deeper while smoking [7]. For these reasons it is likely that concentrations of such harmful chemicals are even higher in mothers who have difficulty quitting smoking while pregnant and that the harmful effects of those chemicals are compounded in their unborn fetuses. Of note in this context is the limitation of our study to assess aqueous smoke extracts only. At the same time, however, the detection of such noteworthy skeletal malformations despite the omission of solvent-dilutable organic compounds, which are in themselves highly toxic, speaks for the problematic association of tobacco smoke exposure with skeletal defects.

We further provide here a potential first insight into why embryotoxicity elicited by the conventional Camel SS smoke is carried by its general (unspecific) cytotoxicity and why Camel Blue SS smoke exposure may instead confer embryotoxicity through authentic chemical impact on developmental processes. While it may be assumed that the chemicals in tobacco smoke solutions trigger signaling cascades that are detrimental to development, another potential mechanism of action is that they induce mild oxidative stress. As we show here, the developmental toxicity of the harm-reduction tobacco product seems defined by its ability to create ROS at levels that are insufficient to kill the cells. As recently put forth by Hansen and Harris [59], teratogenesis is defined by dysmorphogenetic events that may precede excessive cell death. While cytotoxicity focuses on the accumulation of ROS, the impediment of antioxidant capacities, and consequent cell death, developmental toxicity may be the result of untimely regulation of critical cellular signaling rather than the result of early cell death. Our results outlined here offer support for this notion in that while Camel SS and Camel Blue SS effective doses both demonstrated reduced osteogenesis and upregulation of pro-apoptotic gene expression, Camel Blue SS effective cultures specifically failed to achieve complete caspase cascade activation and cell death as seen in Camel SS effective doses. It follows, then, that additional molecular or cellular players may be involved in the differential outcomes mediated by different tobacco products. 

Along this argumentation, disruption of normal tissue redox balances has also been reported to interfere with normal biological processes that can lead to pathological outcomes including DNA damage [60,61,62]. Given that evidence of DNA damage was exclusively observed in cultures exposed to Camel SS effective doses, our results suggest that elevated ROS could cause a DNA damage-mediated mode of action for embryotoxicity outcomes following Camel but not Camel Blue exposure. However, this notion was not supported by experimental evidence, as antioxidant treatment did not reduce DNA damage cell populations in Camel SS effective doses. Nonetheless, given that DNA damage is well-reported to activate caspase 9-mediated apoptosis [63,64,65], it follows that concurrent *CASP9* mRNA upregulation and posttranslational activation of caspase 9 protein in Camel SS effective dose cultures support a biochemical basis for intrinsic apoptotic responses observed exclusively with Camel exposure. 

During the intrinsic apoptosis process, caspase 9 has been reported to regulate the collapse of mitochondrial membrane potential, causing further mitochondrial disruption [66,67]. Aberrant mitochondrial behavior is well-associated with stress responses and a variety of pathologies [68,69], and also reported to follow mitochondrial membrane depolarization and precede mitophagy and apoptosis [60,70]. In accordance with elevated caspase 9 activity, our study found concurrent functional and morphological mitochondrial changes exclusively in Camel effective dose cultures that were reversed when caspase 9 was inhibited. Indeed, BAX, transcripts for which were exclusively up-regulated in Camel SS ED cells, may be activated by DNA damage [71] and can form a channel in the mitochondrial membrane that eventually mediates cytochrome C release [72,73], a notable outcome of Camel SS exposure. Through cytochrome C release BAX helps to assemble the apoptosome from APAF1 and CASP9, which activates and cleaves CASP3 downstream, ultimately executing cell death [74], another noted outcome of Camel SS exposure. The reverse relationship has also been described where BAX activation is CASP9 dependent [75]. This context provides further support for the notion that DNA damage likely elicited the caspase 9-driven cytotoxic responses that may be responsible for osteogenic inhibition following SS Camel exposure. 

Mitochondrial morphological changes observed in the absence of altered mitochondrial function or DNA damage in Camel Blue SS smoke effective dose cultures hint at divergent modes of embryotoxic action for the conventional versus the harm-reduction tobacco product of this brand. In point of fact, the mitochondria and the endoplasmic reticulum (ER) have an important crosstalk relationship that may be of further relevance in the context of development and this study [76,77,78,79,80]. In contrast with Camel SS ED cultures, Camel Blue SS ED cells demonstrated upregulation of caspase 4 mRNA and protein with only little caspase 9 activation. Notably, caspase 4 has been proposed to function as an ER-stress specific caspase [37,81,82,83], not least because it mainly localizes to the ER [81]. Given that treatment with caspase 4 rescued mitochondrial network morphology, it is possible that ER stress was also involved in the differential molecular response of cells exposed to the Camel Blue effective dose. Notably, disruption to normal crosstalk between the mitochondria and ER has been implicated in developmental, metabolic and degenerative diseases [77,78,79,80]. Moreover, ER stress can interfere with protein folding processes [84], which may be to the detriment of osteogenic differentiation as new sets of proteins required for differentiation progression may be made incorrectly or not at all. Secretion of critical regulatory proteins by osteoblasts and their precursors could also be adversely impacted in this context. Follow up studies, however, are required to confirm ER stress and dysfunction in Camel Blue exposed cultures.

In COS-7 cells, overexpression of caspase-4 induces activation of the death protease CASP3 [85]. Conversely, biochemical and genetic evidence suggests that inhibition of CASP4 ablates ER stress-induced apoptosis [86,87,88]. Why, in our study then, CASP4 activation does not lead to CASP3 activation in CB SS cells remains nebulous, but it may be speculated that CASP4 plays additional roles in processes other than apoptosis. In support of this notion, there is evidence in the literature to suggest a link between CASP4, IL10, one of the six unique transcripts found to be regulated in Camel Blue SS ED exposed cells, and inflammation. For instance, CASP4 mediates non-canonical activation of the NLRP3 inflammasome in human myeloid cells [89]. Further, it has been proposed that CASP4 may function mainly via the NFκB signal pathway in inflammatory responses, leading to NFκB-dependent transcriptional up-regulation and secretion of important cytokines and chemokines [90]. While many of the NFκB targets are classical pro-inflammatory cytokines, the NF-κB family member p50 is also known to promote transcription of the anti-inflammatory IL-10 [91,92]. 

Aside from its anti-inflammatory properties, IL-10 exhibits osteoblastogenic or osteolytic attributes, depending on dosage. While IL-10-deficient mice exhibit reduced bone formation [93] and low physiological concentrations of IL-10 induce osteoblastogenesis by activating the p38 MAPK signaling pathway in human MSCs, higher pathological doses of IL-10 inhibit osteoblastogenesis by activating NFκB signaling [94]. These data corroborate the notion that the Camel Blue SS mediated increase in IL-10 may underlie the noted phenotypic alterations in our cultures. 

Aside from the CASP4/IL-10 axis, a second potential mechanism for the dampened osteogenic differentiation efficiency can be construed from the known relationship between IL-10 and Fas ligand, the transcript for which was also uniquely regulated upon Camel Blue SS exposure. IL-10 production downstream of Fas ligand-mediated engagement of the Fas receptor represents an important anti-inflammatory mechanism in monocytes [95] that occurs in the absence of executioner caspase activation and thus apoptosis. Again, it may be through IL-10 that osteogenic differentiation is inhibited. In addition, IL-10 may protect cells from Fas-induced apoptosis by dampening CASP8 activation, as is the case in intestinal epithelial cells [96], which may be the reason for the lower CASP8 response in Camel Blue SS ED we observe. The enhanced expression of *TNFSRF1B* in Camel Blue SS may additionally tip the balance towards survival, not apoptosis, by activating AKT and NFκB [97]. 

Supplementarily, the involvement of Fas may also have direct consequences for osteogenic cell fate specification. Osteoblasts constitutively express FAS and FASLG [98,99] and gld mice, which have a defect in the FASLG-mediated apoptotic pathway, have increased bone trabecular volume in vivo [100]. Mice without functional Fas-ligand also have an increased recruitment of cells of mesenchymal origin and an abnormal pattern of differentiation and maturation of the newly formed mesenchymal tissues [101]. In reverse, this would imply that overactivation of FASLG may decrease proliferation of bone progenitors and total volume of newly formed bone, which is what we find here, and a failure to recruit mesenchymal progenitor cells. Taken together, independently of how IL-10, FASLG and CASP4 specifically interact, the increase in IL-10 may therefore underlie the phenotypic alterations noted in the Camel Blue SS mediated osteogenic defect.

## 5. Conclusions

The results from this study imply a causal link between the tobacco-induced osteogenic differentiation effect and oxidative stress. However, observed differential mRNA upregulation patterns between Camel SS and Camel Blue SS suggested that a precise molecular interplay may be responsible for ultimate phenotypic outcomes following exposure to a specific tobacco product. As follows, this result also implies that while Camel SS and Camel Blue SS actively harm differentiating osteoblasts, both products may act distinctly upon cellular regulatory mechanisms. Conceptually, our finding remains important despite global tobacco education programs that generally caused smoking to decline. This is because approximately 10–20% of women in the United States still smoke during pregnancy and home exposure to secondhand smoke is prevalent amongst pregnant women in low- and middle-income households [2].

## Data Availability

The data presented in this study are available in the article and supplementary material.

## Supplementary Materials

The following supporting information can be downloaded at: https://www.mdpi.com/article/10.3390/antiox11122474/s1, Figure S1: Superoxide anion content measured upon reaction of the cells with luminol in response to antioxidant treatment.
