# Supplementary material for: Sidestream Smoke Extracts from Harm-Reduction and Conventional Camel Cigarettes Inhibit Osteogenic Differentiation via Oxidative Stress and Differential Activation of intrinsic Apoptotic Pathways
Source: Antioxidants (Basel). 2022 Dec 15;11(12):2474. doi: 10.3390/antiox11122474 (PMC9774253; doi:10.3390/antiox11122474)
Supplement: Supplementary file 1 [file antioxidants-11-02474-s001.zip › antioxidants-2056460-supplementary.pdf]

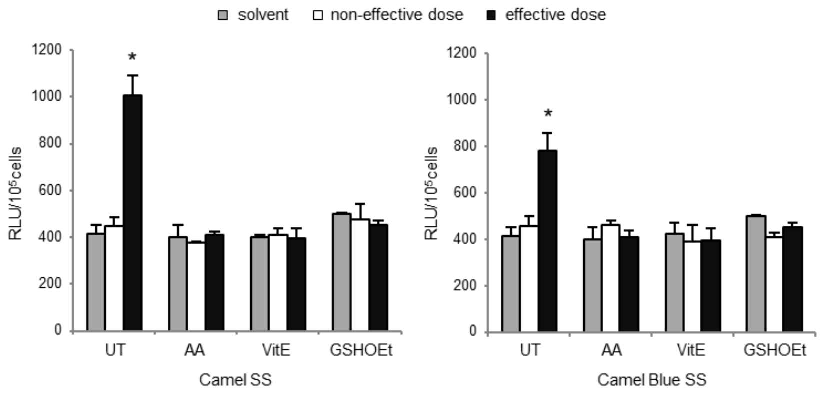

Supplemental Figure S1: Superoxide anion content measured upon reaction of the cells with luminol in response to antioxidant treatment; n = 3±SD, * $p < 0.05$ One-Way ANOVA versus untreated cultures. AA, ascorbic acid; GSHOEt, glutathione reduced ethyl ester; RLU, relative light unit; UT, untreated solvent control; VitE, Vitamin E.
